# ATP-Dependent Persister Formation in *Escherichia coli*

**DOI:** 10.1128/mBio.02267-16

**Published:** 2017-02-07

**Authors:** Yue Shan, Autumn Brown Gandt, Sarah E. Rowe, Julia P. Deisinger, Brian P. Conlon, Kim Lewis

**Affiliations:** Department of Biology, Antimicrobial Discovery Center, Northeastern University, Boston, Massachusetts, USA; Indiana University Bloomington

## Abstract

Persisters are dormant variants that form a subpopulation of cells tolerant to antibiotics. Persisters are largely responsible for the recalcitrance of chronic infections to therapy. In *Escherichia coli*, one widely accepted model of persister formation holds that stochastic accumulation of ppGpp causes activation of the Lon protease that degrades antitoxins; active toxins then inhibit translation, resulting in dormant, drug-tolerant persisters. We found that various stresses induce toxin-antitoxin (TA) expression but that induction of TAs does not necessarily increase persisters. The 16S rRNA promoter *rrnB* P1 was proposed to be a persister reporter and an indicator of toxin activation regulated by ppGpp. Using fluorescence-activated cell sorting (FACS), we confirmed the enrichment for persisters in the fraction of *rrnB* P1*-gfp* dim cells; however, this is independent of toxin-antitoxins. *rrnB* P1 is coregulated by ppGpp and ATP. We show that *rrnB* P1 can report persisters in a *relA*/*spoT* deletion background, suggesting that *rrnB* P1 is a persister marker responding to ATP. Consistent with this finding, decreasing the level of ATP by arsenate treatment causes drug tolerance. Lowering ATP slows translation and prevents the formation of DNA double-strand breaks upon ﬂuoroquinolone treatment. We conclude that variation in ATP levels leads to persister formation by decreasing the activity of antibiotic targets.

## IMPORTANCE

Persisters are a subpopulation of antibiotic-tolerant cells responsible for the recalcitrance of chronic infections. Our current understanding of persister formation is primarily based on studies of *E. coli*. The activation of toxin-antitoxin systems by ppGpp has become a widely accepted model for persister formation. In this study, we found that stress-induced activation of mRNA interferase-type toxins does not necessarily cause persister formation. We also found that the persister marker *rrnB* P1 reports persister cells because it detects a drop in cellular ATP levels. Consistent with this, lowering the ATP level decreases antibiotic target activity and, thus, leads to persister formation. We conclude that stochastic variation in ATP is the main mechanism of persister formation. A decrease in ATP provides a satisfactory explanation for the drug tolerance of persisters, since bactericidal antibiotics act by corrupting energy-dependent targets.

## INTRODUCTION

Chronic infections are caused mainly by drug-susceptible pathogens but are difficult to eradicate (1). This is particularly true for biofilms, microbial communities that form on indwelling devices or within soft tissues and are protected from the immune system by a layer of exopolymers (2, 3). An increasing body of evidence points to persister cells as the main culprit of drug tolerance. Produced stochastically by all pathogens studied, persisters are multidrug-tolerant phenotypic variants of the wild type (4, 5). *In vivo* studies have shown that *Escherichia coli* bacteria causing urinary tract infections (UTIs) form drug-tolerant biofilms within bladder epithelial cells (6, 7). Mutants with elevated levels of persisters (high persister mutants) are common among *Pseudomonas aeruginosa* isolates from patients with cystic fibrosis (8), among *Mycobacterium tuberculosis* isolates from patients with tuberculosis (9), and among *Candida albicans* from patients with oral thrush (10). Persisters were reported in a chronic *Staphylococcus aureus* mouse infection model (11). These observations link persisters to clinical manifestation of chronic disease.

Most of what we know about the mechanism of persister formation comes from the study of *E. coli*, and toxin-antitoxin (TA) modules have been linked to persister formation. It has been shown that stochastic expression of the HipA kinase, a type II toxin which inhibits protein synthesis by phosphorylating Glu-tRNA synthetase (12, 13), contributes to the formation of persisters (12, 14). Gain of function mutations in *hipA* produce elevated levels of persisters *in vitro*, and the same mutants are present in patients with relapsing UTI (14). Stress-induced toxin expression has also been linked to increases in drug tolerance. Fluoroquinolone antibiotics kill cells by stabilizing toxic reaction intermediates, such as double-stranded breaks in DNA generated by DNA gyrase and topoisomerase (15). The main function of SOS is to express DNA repair enzymes, but the same regulatory pathway also turns on production of the type I TisB toxin in a subpopulation of *E. coli* cells (16). TisB forms an ion channel in the cytoplasmic membrane, decreasing proton motive force and ATP levels, which leads to drug tolerance (16, 17).

One important class of toxins that are linked to persisters are the mRNA interferases encoded by type II TA loci (18). Transcriptome analysis revealed that persisters express high levels of mRNA interferases (19, 20). Ectopic expression of these toxins causes multidrug tolerance (19). Stochastic overexpression of the *yoeB* toxin in individual cells has been reported to protect from ampicillin as well (21). Deleting single interferases does not produce a phenotype in *E. coli* (22), but the level of persisters was reported to be drastically decreased in a strain with deletions of 10 mRNA interferases (Δ10TA) (23). Stress in the form of starvation has also been linked to the expression of mRNA interferases (24). Specifically, the formation of ppGpp by the stringent response has been reported to cause an increase in persisters in *E. coli* and *P. aeruginosa* (25, 26). A recent study showed that ppGpp induces persister formation through the activation of mRNA interferases and linked stress response, toxin-antitoxin systems, and persister formation (21). The induction of mRNA interferases by stress has become a widely accepted model for persister formation in *E. coli* (27–31).

In this study, we found that most stresses induce mRNA interferase expression, but this leads to an increase in the persister level only in the case of the stringent response. The known persister reporter *rrnB* P1, the promoter of rRNA, senses both ppGpp and ATP. We found that *rrnB* P1 reports persister levels in Δ10TA and ppGpp^0^ backgrounds, indicating that it is the variation in the level of ATP that determines the formation of persisters. These findings are consistent with our recent study in the Gram-positive pathogen *S. aureus* (32) and indicate a universal role of ATP in the drug tolerance of different species. A drop in ATP decreases the activity of antibiotic targets, providing a simple explanation for the mechanism of drug tolerance and persister formation.

## RESULTS

### mRNA interferases are upregulated by stress.

Certain mRNA interferases are expressed during starvation induced by serine hydroxamate or glucose limitation, and it was suggested that this leads to persister formation (24, 33–36). However, evidence linking the induction of mRNA interferases during stress to persister production is lacking. We sought to determine whether various stresses activate these mRNA interferases and induce persister formation.

We screened 10 mRNA interferases for induction of transcription by several stresses, including acid, high sodium, high osmolarity, isoleucine starvation (stringent response), and phosphate starvation. Since the promoters of the TAs are repressed by free antitoxin or toxin-antitoxin complexes, we reasoned that if a toxin is activated, there should be less antitoxin and higher transcription activity. Thus, activation of the promoter indicates an imbalance between toxin and antitoxin (37). *E. coli* strains from a library of promoter-*gfp* fusions (38) were used for this purpose. Each stress induces a subset of TAs. Isoleucine starvation induces all 10 TAs, followed by osmotic stress (8 TAs), phosphate stress (6 TAs), acid stress (4 TAs), and NaCl stress (1 TA) (Table 1; see also Fig. S1 in the supplemental material). The levels of stresses we chose were reported to represent physiologically relevant conditions and do not significantly impact growth (see Fig. S2). The induction of 10 TA systems by isoleucine starvation agrees with previous studies and validates our approach (34, 39).

10.1128/mBio.02267-16.1 FIG S1  Interferases are differentially upregulated by stress. Interferase promoters driving the expression of *gfp* were assayed for induction in response to various stress conditions (described in detail in Materials and Methods): isoleucine starvation (A), osmotic stress (B), phosphate starvation (C), acid stress (D), and sodium stress (E). GFP fluorescence (excitation at 485 nm and emission at 528 nm) and optical density (OD_600_) were recorded every 30 min for 14 h. Background fluorescence was subtracted using a strain carrying a plasmid with a promoterless GFP. Comparing a stressed strain with a control may be problematic, since stress affects growth rate and the ODs at the same time point may be different. To compare the strains at comparable OD levels, we binned the results into a narrow range of OD measurements. We first set the 0-to-0.1 OD_600_ range as bin 1 and calculated the average GFP values corresponding to that bin. For each stress, we binned the OD values into 0 to 0.1, 0.1 to 0.2, 0.2 to 0.3, 0.3 to 0.4, and 0.4 to 0.5. When too few data points fell within a bin to allow for statistical analysis, 0.2-unit bins were used. Data are the average results from at least three independent experiments. Error bars represent standard deviations. An asterisk indicates a significant (*P* < 0.05 by two-tailed Student’s *t* test) increase in GFP fluorescence under stress compared to nonstress conditions. Download FIG S1, PPT file, 1.3 MB. Copyright © 2017 Shan et al. 2017 Shan et al. This content is distributed under the terms of the Creative Commons Attribution 4.0 International license.

10.1128/mBio.02267-16.2 FIG S2  Effects of stress conditions on growth. (A to E) *E. coli* growth under stress conditions was measured by optical density. MG1655 (wild type [WT]) was grown under an indicated stress condition and the corresponding control condition as described in Materials and Methods. Optical density (OD_600_) was recorded every 30 min. Data are the average results from at least three biological replicates. Error bars represent standard error. Download FIG S2, TIF file, 1.1 MB. Copyright © 2017 Shan et al. 2017 Shan et al. This content is distributed under the terms of the Creative Commons Attribution 4.0 International license.

**TABLE 1:** Toxin-antitoxins are induced in response to stress conditions

Stressor	Promoter activity for^a^:									
	*chpSB*	*dinJ yafQ*	*hicAB*	*higAB*	*mazEF*	*mqsRA*	*prlF yhaV*	*relBE*	*yafNO*	*yefM yoeB*
Isoleucine starvation	+	+	+	+	+	+	+	+	+	+
Osmotic stress		+		+	+	+	+	+	+	+
Phosphate starvation	+	+			+		+		+	+
Acid stress		+				+		+		+
Sodium stress		+								

^a^ Promoters of interferases driving the expression of *gfp* were assayed for induction in response to various stress conditions (described in detail in Materials and Methods). +, promoter activity was significantly (*P* < 0.05 by two-tailed Student’s *t* test) increased by stress as calculated by the method described in the legend to Fig. S1. GFP fluorescence (excitation at 485 nm and emission at 528 nm) and optical density (OD_600_) were recorded every 30 min. At least three independent experiments were performed (*n* ≥ 3).

### Stress-induced TA production plays a limited role in persister formation.

**FIG 1:**
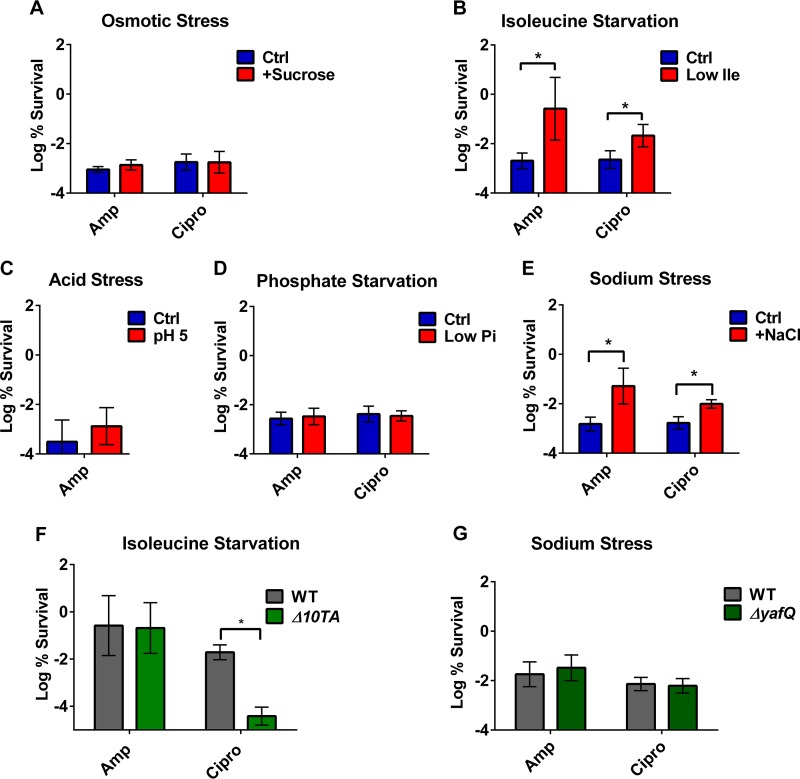
Upregulation of TA modules plays a limited role in persister formation. (A to E) MG1655 (wild type [WT]) was grown under stress as described in Materials and Methods to approximately the same starting density as the control. Cultures were then challenged with ciprofloxacin (0.5 μg/ml) or ampicillin (100 μg/ml) for 4 h (A, C, D, E) or 5 h (B). (F, G) Strains with deletions of toxins showing increased expression under sodium stress or isoleucine starvation were tested for persister formation. MG1655 (WT) and isogenic mutant strains were grown to the same cell density under the indicated stress and then challenged with ampicillin (100 μg/ml) or ciprofloxacin (0.5 μg/ml) for 5 h (F) or 4 h (G). Results are expressed as percent survival by comparison to untreated culture prior to the addition of antibiotic. Data are the average results from at least two independent experiments performed with three biological replicates (*n* ≥ 6). An asterisk indicates a significant difference (*P* < 0.05) by two-tailed Student’s *t* test. Error bars represent standard deviations.

Next, we tested whether stresses induce persister formation. Surprisingly, of the 5 stresses we tested, only isoleucine starvation and NaCl stress increased the level of persisters (Fig. 1B and E). Phosphate starvation, acid stress, and osmotic stress, in spite of strong induction of several TAs, had no effect on drug tolerance (Fig. 1A, C, and D). These results indicate that the activation of mRNA interferases does not necessarily increase persisters.

We next tested whether the increased persister formation due to isoleucine starvation or NaCl stress was dependent on the induction of TA modules. Only one toxin, *yafQ*, is upregulated upon NaCl stress. The deletion of *yafQ* does not change the level of persisters induced by sodium stress (Fig. 1F). For isoleucine starvation, since all 10 toxins are upregulated, we used the Δ10TA strain constructed by the Gerdes group (23) to examine its effect on tolerance. In the Δ10TA strain, isoleucine starvation induced persisters tolerant to ampicillin but failed to induce persisters tolerant to ciprofloxacin (Fig. 1G). Overall, it appears that mRNA interferases play a limited role in stress-induced persister formation. We next decided to reexamine the role of these TAs in the stochastic formation of persisters in an unstressed culture.

We first tested persister levels of the *E. coli* Δ10TA strain under the same conditions as reported by Maisonneuve et al. (23) and found that the strain has a lower level of persisters surviving treatment with ciprofloxacin and ampicillin (see Fig. S3A and B in the supplemental material), in general agreement with previous studies. However, under other conditions we tested, such as minimal medium or minimal medium supplemented with amino acids, Δ10TA only showed a decreased level of persisters tolerant to ciprofloxacin, and not to ampicillin (see Fig. S3C to F).

10.1128/mBio.02267-16.3 FIG S3  Persister levels of Δ10TA under different conditions. Exponentially growing cells of MG1655 (WT) and of the isogenic interferase deletion strain (Δ10TA) in different growth media were exposed to either ampicillin (100 µg/ml) or ciprofloxacin (1 µg/ml) as indicated. At time zero, an aliquot of a culture was diluted and plated for CFU. At each subsequent time point, an aliquot of the culture was washed and plated to quantify persisters. Data are the average results from at least two independent experiments performed with three biological replicates (*n* ≥ 6). Error bars represent standard deviations. Download FIG S3, TIF file, 1.4 MB. Copyright © 2017 Shan et al. 2017 Shan et al. This content is distributed under the terms of the Creative Commons Attribution 4.0 International license.

mRNA interferases were linked to persister formation because ectopic overexpression of these genes leads to rapid degradation of mRNA and shutdown of translation (18). It has been assumed that the same mechanism, inhibition of translation, is responsible for persister formation due to stochastic expression of toxins (40). Consistent with this, it has been shown that translation inhibition leads to increased persisters (41). We decided to test this conclusion experimentally and reasoned that if mRNA interferases act by inhibiting protein synthesis, then the Δ10TA strain should have the same level of persisters as the wild type in the presence of an inhibitor of translation. For this, we pretreated cells with chloramphenicol or tetracycline to shut down translation. As expected, inhibition of translation increases the formation of persisters tolerant to both antibiotics in the wild type (see Fig. S4A and B in the supplemental material). However, Δ10TA still forms fewer persisters tolerant to ciprofloxacin than does the wild type in the presence of chloramphenicol or tetracycline (see Fig. S4B). The degree of persister decrease in Δ10TA is actually similar irrespective of the presence or absence of translation inhibitors. These results indicate that the deletion of 10 TAs decreases the level of persisters tolerant to fluoroquinolones through a translation-independent mechanism.

10.1128/mBio.02267-16.4 FIG S4  Induction of persisters by toxins does not depend on inhibition of translation. MG1655 (WT) and the isogenic TA deletion strain (Δ10TA) were grown to exponential phase in LB medium and exposed to 100-µg/ml ampicillin (A) or 1-µg/ml ciprofloxacin (B). For inhibition of translation, cultures were incubated with 100-µg/ml chloramphenicol or 20-µg/ml tetracycline for 45 min before antibiotic exposure (right). Results are expressed as percent survival by comparison to untreated culture prior to the addition of antibiotic. Data are the average results from at least two independent experiments performed with three biological replicates (*n* ≥ 6). Error bars represent standard deviations. An asterisk indicates a significant difference (*P* < 0.05) by two-tailed Student’s *t* test. Download FIG S4, TIF file, 0.7 MB. Copyright © 2017 Shan et al. 2017 Shan et al. This content is distributed under the terms of the Creative Commons Attribution 4.0 International license.

We considered that the Δ10TA strain may have accumulated some pleiotropic mutations during the sequential deletion process. We sequenced the genomes of Δ10TA and the parental MG1655 strain. We found 160 single-nucleotide polymorphisms (SNPs) in Δ10TA, and 79 of these SNPs cause amino acid substitutions (see Data Set S1 in the supplemental material). Many of these SNPs are located in prophage loci, but no obvious changes that could result in pleiotropic effects were observed. How exactly mRNA interferases affect persister formation remains to be determined.

10.1128/mBio.02267-16.5 DATA SET S1  SNPs in the Δ10TA strain in comparison to the isogenic MG1655 (WT) strain. Download DATA SET S1, XLSX file, 0.03 MB. Copyright © 2017 Shan et al. 2017 Shan et al. This content is distributed under the terms of the Creative Commons Attribution 4.0 International license.

### Toxin-activating components.

It is widely accepted that starvation induces ppGpp synthesis by RelA/SpoT, the alarmone inhibits the PPX phosphatase, increased levels of polyphosphate activate the Lon protease that degrades the antitoxins, and active toxins are released, inhibiting translation, which causes drug tolerance (21).

It has been known that Lon protease is required for the survival of cells treated with fluoroquinolones (42). Ciprofloxacin damages DNA, which induces the SOS response and expression of the cell division inhibitor SulA. When DNA is repaired, SulA is degraded by Lon and cell growth resumes. In a *lon* mutant, SulA accumulates, leading to cell elongation and eventual lysis and death (42). Deletion of *lon* alone decreases survival under exposure to ciprofloxacin significantly (see Fig. S5B in the supplemental material), likely due to SulA accumulation rather than antitoxin accumulation. A *lon sulA* double deletion mutant is therefore required to test the effects of Lon on antitoxin degradation (43). In our hands, mutants with deletions of the upstream genes proposed to control toxin activity, including the *lon* protease (in a *sulA* deletion background), the *ppx* phosphatase, the *ppk* polyphosphate kinase, and Δ*ppx* Δ*ppk*, have the same level of persisters as the wild-type strain (see Fig. S5C). These results are consistent with several recent reports that neither *lon* (43, 44) nor *ppk* and *ppx* (45) play a role in persister formation.

10.1128/mBio.02267-16.6 FIG S5  Lon and PolyP do not impact multidrug tolerance. (A, B) MG1655 (WT) and isogenic Δ*lon* and Δ*lon* Δ*sulA* strains were grown to approximately the same starting CFU and exposed to either 100-µg/ml ampicillin or 1-µg/ml ciprofloxacin as indicated. At time zero, an aliquot was diluted and plated for CFU count. At each subsequent time point, an aliquot of the culture was washed and plated to recover persisters. (C) MG1655 (WT) and isogenic Δ*ppx*, Δ*ppk*, and Δ*ppx* Δ*ppk* strains were grown to approximately the same starting CFU and exposed to either 100-µg/ml ampicillin or 1-µg/ml ciprofloxacin, as indicated, for 4 h. Results are expressed as percent survival by comparison to untreated culture prior to the addition of antibiotic. Data are the average results from at least two independent experiments performed with three biological replicates (*n* ≥ 6). Error bars represent standard deviations. An asterisk indicates that the CFU count was lower than the limit of detection. Download FIG S5, TIF file, 0.5 MB. Copyright © 2017 Shan et al. 2017 Shan et al. This content is distributed under the terms of the Creative Commons Attribution 4.0 International license.

### *rrnB* P1 reports persister formation independently of mRNA interferases and ppGpp.

In a growing population, the majority of cells have high levels of expression of 16S RNA, controlled by the *rrnB* P1 promoter. Cells with an inactive *rrnB* P1 promoter are likely dormant, and sorting out dim cells of a strain expressing degradable green fluorescent protein (GFP) under the control of this promoter resulted in the isolation of persisters (20). In an independent study, RpoS-mCherry was used as a marker for ppGpp to identify persisters (21). ppGpp activates RpoS transcription and inhibits its proteolysis (46). Bright RpoS-mCherry cells did not grow and were not killed by ampicillin (21). In the same study, it was also shown that cells with high expression of RpoS-mCherry had low levels of *rrnB* P1-*gfp*^unstable^. *rrnB* P1 is repressed by ppGpp (47), and it was concluded that low *rrnB* P1/high RpoS enable the identification of persisters by indicating increased levels of ppGpp that would lead to high expression of toxins.

**FIG 2:**
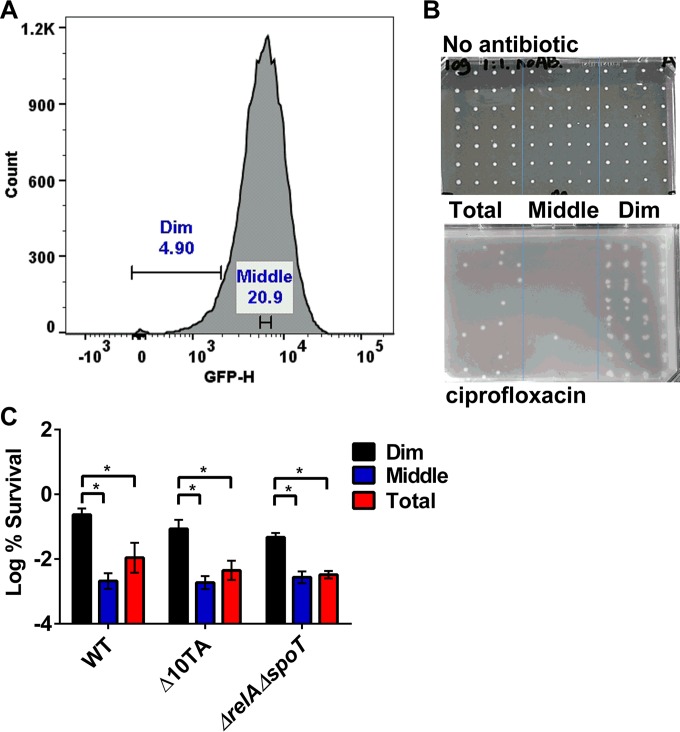
*rrnB* P1 promoter activity correlates with persisters independently of mRNA interferases and ppGpp. (A) Exponentially growing cells of MG1655-ASV carrying an *rrnB* P1::*gfp*^unstable^ transcription fusion were exposed to 1-µg/ml ciprofloxacin for 4 h. The antibiotic-treated cells were then analyzed by FACS. The dim (5%), middle (20%), and total (100%) fractions of the population were isolated by cell sorting. Cells from the dim, middle, and total fractions of the population were sorted onto agar plates, and the persisters were quantified by CFU. (B) A representative plate image after sorting. (Top) One cell was sorted onto each spot from an exponentially growing culture without antibiotic challenge. (Bottom) One thousand cells were sorted onto each spot from a ciprofloxacin-challenged culture. (C) MG1655-ASV (WT), isogenic Δ10TA, and Δ*relA* Δ*spoT* cultures were exposed to 1-µg/ml ciprofloxacin for 4 h and underwent FACS analysis and cell sorting. The percent survival for each fraction was determined by comparing the CFU count with the total number of sorted cells. Data are the average results from at least two independent experiments performed with three biological replicates (*n* ≥ 6). Error bars represent standard deviations. An asterisk indicates a significant difference (*P* < 0.05) by two-tailed Student’s *t* test.

We sorted dim cells with a low level of *rrnB* P1 transcription and measured their survival (Fig. 2A and B). The dim population was ~100-fold enriched in persisters surviving killing by ciprofloxacin compared to the level in the bulk of the population (Fig. 2C), consistent with the previous study (20).

In order to test the dependence of persisters obtained by sorting *rrnB* P1-*gfp*^unstable^ cells on toxins, we constructed a strain carrying this reporter in the background of Δ10TA. Surprisingly, we found that in a Δ10TA background, dim cells carrying *rrnB* P1-*gfp*^unstable^ are similarly enriched in persisters (Fig. 2C). This result suggests that *rrnB* P1 reports persisters independently of mRNA interferases. It appears that activation of toxins is not responsible for the drug tolerance of cells with low levels of *rrnB* P1 expression. We then sought to determine the nature of the link between persisters and the activity of the *rrnB* P1 promoter.

We first determined whether *rrnB* P1-reported persister formation is controlled by ppGpp. To test this, we constructed an *rrnB* P1-*gfp*^unstable^ Δ*relA* Δ*spoT* strain. Deletion of *relA*/*spoT* leads to a much longer lag phase and lower final CFU count in stationary phase (see Fig. S6 in the supplemental material). We grew the *rrnB P1-gfp*^unstable^ Δ*relA* Δ*spoT* strain to the same CFU count as the wild type and compared the survival of the dim 5% of cells with that of the middle 20% and the total population. The *rrnB* P1*-gfp*^unstable^ Δ*relA* Δ*spoT* dim population was enriched in persisters similarly to the wild-type (Fig. 2C). These results indicate that *rrnB* P1 may report persister levels in the absence of ppGpp.

10.1128/mBio.02267-16.7 FIG S6  MG1655 Δ*relA* Δ*spoT* has a longer lag phase and lower cell density in stationary phase. Overnight cultures of MG1655 (WT) and an isogenic Δ*relA* Δ*spoT* mutant were diluted 1:100 into LB medium, and growth was monitored by plating for CFU counts. Data are the average results from at least two independent experiments performed with three biological replicates (*n* ≥ 6). Error bars represent standard deviations. Download FIG S6, TIF file, 0.4 MB. Copyright © 2017 Shan et al. 2017 Shan et al. This content is distributed under the terms of the Creative Commons Attribution 4.0 International license.

**FIG 3:**
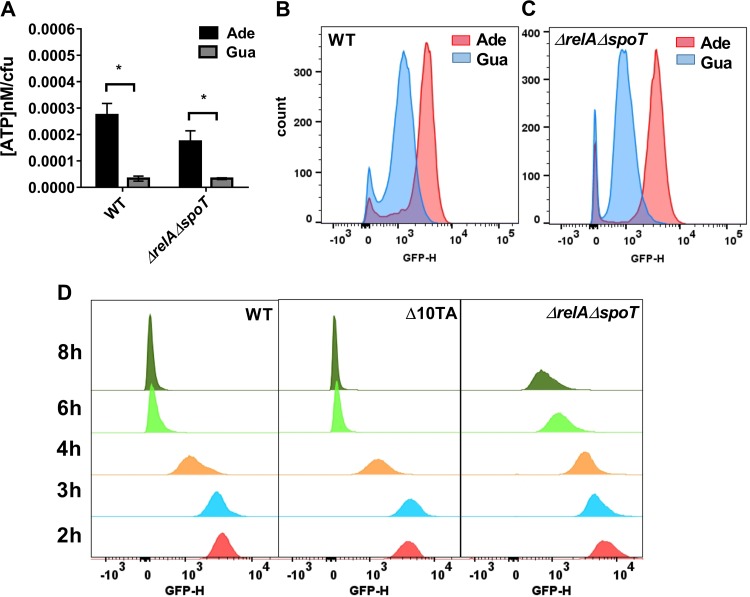
*rrnB* P1 senses ATP level independently of ppGpp and is repressed upon entrance into stationary phase. (A) Overnight cultures of ASV *ΔpurE* (WT) and an isogenic ASV Δ*purE* Δ*relA* Δ*spoT* (Δ*relA* Δ*spoT*) mutant were diluted 1:100 into MOPS minimal medium supplemented with 0.2% glucose, 0.2% Casamino acid, and 10-μg/ml thiamine and with either adenine or guanine (0.2 mM), as indicated. Cells were grown for 2 h for the WT and 3 h for the Δ*relA* Δ*spoT* mutant before measuring ATP level and GFP fluorescence. Data are the average results from two independent experiments performed with three biological replicates (*n* = 6). Error bars represent standard deviations. An asterisk indicates a significant difference (*P* < 0.05) by two-tailed Student’s *t* test. (B, C) GFP fluorescence was analyzed by FACS to determine the transcription level of *rrnB* P1. (D) Stationary-phase *E. coli* MG1655-ASV (WT) and isogenic Δ10TA and Δ*relA* Δ*spoT* cultures were diluted 1:100 into LB medium. At each time point, GFP fluorescence was analyzed by FACS to determine the transcription activity of *rrnB* P1.

Interestingly, apart from being repressed by ppGpp, *rrnB* P1 was reported to be induced by nucleotide triphosphates, in particular by ATP (48). rRNA promoters (*rrn* P1) form unusually short-lived complexes with RNA polymerase and require much higher NTP concentrations for transcription initiation than mRNA promoters (49). This makes *rrnB* P1 a sensitive ATP reporter. We reasoned that a drop in ATP may cause persister formation, which is reported by the decrease in *rrnB* P1 expression. By adding exogenous adenine or guanine to purine auxotrophs, as previously described (48), we were able to raise the cellular ATP concentration (Fig. 3A) and saw a corresponding shift in *rrnB* P1 expression in both the wild type and a Δ*relA* Δ*spoT* strain (Fig. 3B and C). This confirmed that *rrnB* P1 is an ATP sensor which can act independently of ppGpp.

Bactericidal antibiotics kill by corrupting active targets that require ATP (50–52). ATP levels are decreased in stationary phase, and as the culture density increases, the level of persisters rises, reaching 1% once growth ceases (53). Of note is that ATP was proposed to play an important role in the regulation of *rrnB* P1 expression upon transition from exponential into stationary state (47). Analysis of *rrnB* P1-*gfp*^unstable^ fluorescence over time showed that the fraction of dim cells progressively increases, reaching a maximum at stationary state (Fig. 3D). A similar result was observed in a Δ10TA background, showing that toxin-antitoxins do not affect the activity of the *rrnB* P1 promoter. In a Δ*relA* Δ*spoT* background, the decrease in *rrnB* P1 activity as the culture progresses from early exponential into stationary state is still obvious, but the transition is delayed and the cells are brighter than in the wild type or the Δ10TA strain. This probably reflects the long lag phase of the strain (see Fig. S6 in the supplemental material) and the role of ppGpp in controlling this promoter. Given that the *rrnB* P1 promoter is an ATP sensor, these results suggest that the dim population is enriched in persisters due to low ATP levels. Persisters in a growing population appear to be the dim cells that entered early into a stationary-like phase, which has lower levels of ATP (54, 55).

### A drop in intracellular ATP causes persister formation by decreasing antibiotic target activity.

**FIG 4:**
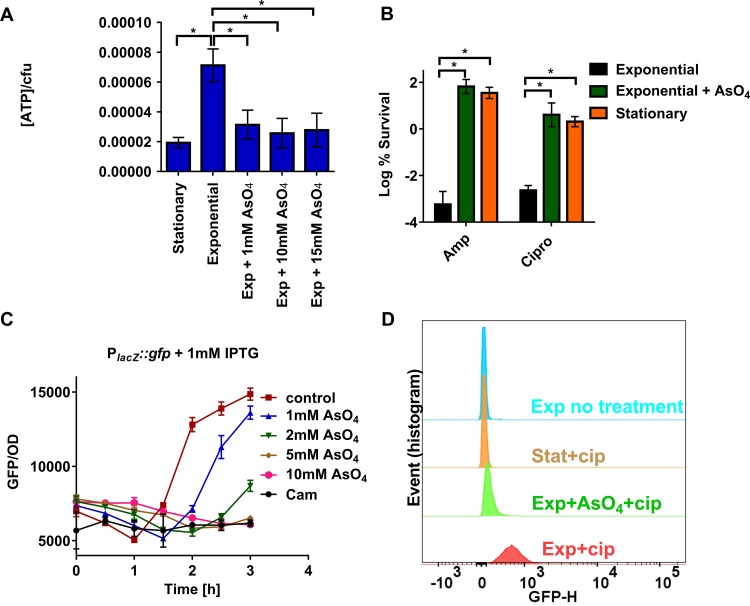
Drop in intracellular ATP level leads to increased persister formation through lowering target activity. (A) ATP levels were measured in stationary and exponentially growing MG1655 (WT). Cells were treated with arsenate for 30 min where indicated. (B) MG1655 culture was grown in LB medium to either exponential phase or stationary phase and then treated with 10 mM arsenate (+AsO_4_) for 30 min where indicated and challenged with ampicillin or ciprofloxacin for 4 h. Results are expressed as percent survival by comparison to untreated culture prior to the addition of antibiotic. Data are the average results from at least two independent experiments performed with three biological replicates (*n* ≥ 6). Error bars represent standard errors. An asterisk indicates a significant difference (*P* < 0.05) by two-tailed Student’s *t* test. (C) *E. coli* MG1655 strain harboring a plasmid-borne P_*lacZ*_::*gfp* fusion was grown to exponential phase. Cultures were pretreated with different concentrations of arsenate or chloramphenicol (Cam) for 30 min. Isopropyl-β-d-thiogalactopyranoside (IPTG) (1 mM) was then added where indicated to induce expression of GFP from the *lacZ* promoter (time zero). GFP fluorescence (excitation at 485 nm and emission at 528 nm) was measured every 30 min. Data points are the average results from the experiment performed in triplicate (*n* = 3). Error bars represent standard deviations. (D) TUNEL assay for DNA fragmentation measurement. *E. coli* MG1655 strain was grown to either exponential phase or stationary phase, and cultures were pretreated with arsenate for 30 min where indicated. Cells were then treated with 1-μg/ml ciprofloxacin for 3 h. No ciprofloxacin was added to the no-treatment control. Cells were then used for TUNEL assay analysis. Free 3′-end DNA was labeled with FITC-dUTP, and FITC intensity was measured by FACS.

We next sought to determine whether lowering the ATP level in an exponential-phase culture will increase the level of persisters. For this, we emulated the stationary level of ATP in a growing culture by depleting ATP with arsenate (Fig. 4A). Cells that had a stationary-like level of ATP tolerated ciprofloxacin and ampicillin similarly to stationary cultures (Fig. 4B). This suggests that a drop in intracellular ATP concentration can indeed cause persister formation and a decrease in ATP is the likely reason for increased persisters in stationary phase.

A decrease in ATP appears to be a satisfactory explanation for multidrug tolerance, as most conventional antibiotics kill by corrupting ATP-dependent targets. A well-documented case of tolerance due to low target activity is the ineffectiveness of β-lactams against stationary-phase cells (50). Upon a decrease in ATP, cell wall synthesis stops, leading to β-lactam tolerance and cessation of growth. We next examined whether the same rationale holds for other antibiotics. We tested whether the translation rate drops in growing cells in which ATP has been decreased to the stationary level by treatment with arsenate. Plasmid-borne inducible P_*lacZ*_::*gfp* was used to measure GFP fluorescence as an indication of the translation rate. It has previously been shown that the rate of translation drops by an order of magnitude in stationary-phase cells (56). Consistent with this, the translation rate dropped dramatically in exponential-phase cells with a stationary-like level of ATP (Fig. 4C). A decrease in the rate of translation explains tolerance to bactericidal inhibitors of protein synthesis, such as aminoglycosides (51).

Fluoroquinolones kill by stabilizing the DNA cleavage complex formed by gyrase and topoisomerase IV, causing DNA double-strand breaks. We found that decreasing ATP to stationary levels promotes the survival of cells treated with ciprofloxacin (Fig. 4B), probably due to a decrease in the activity of the targeted process, which would lead to less DNA fragmentation. To test this, we used the terminal deoxynucleotidyltransferase-mediated dUTP-biotin nick end labeling (TUNEL) assay to measure the amount of free 3′-end DNA fragments (57). We found that pretreating with arsenate protects cells from fluoroquinolone-induced DNA fragmentation (Fig. 4D). Similarly, stationary-phase cells exhibit much lower DNA fragmentation than exponential-phase cells (Fig. 4D). These results explain the tolerance of low-ATP cells to fluoroquinolones.

## DISCUSSION

Persisters are formed through redundant mechanisms; screens of knockout libraries in several species have not produced a strain that does not form persisters (22, 58–61). TA modules have emerged as a major component responsible for persister formation in *E. coli*. *hipA* was the first gene to be linked to persisters, identified in a screen for *hip* mutants in the 1980s (62). A deletion in the *hipBA* TA locus, however, produced no phenotype, and this line of inquiry was largely abandoned. With a resurgence of interest in drug tolerance, gain-of-function *hipA* mutants became a convenient and widely used model to study persisters (63). We recently reported that *hipA* mutants conferring 100- to 1,000-fold increases in persisters are present both in commensals and in clinical isolates from patients with UTI, showing that the HipA toxin in these strains becomes not only biologically relevant but also the main component responsible for persister formation (14). The ability of ectopically expressed interferase (mRNA endonuclease) toxins to produce a similarly large increase in persisters seemed to provide a satisfactory corollary to HipA. Several lines of additional evidence pointed to a role of mRNA interferases in drug tolerance of *E. coli*, including increased expression in isolated persisters, time-lapse microscopy of cells surviving antibiotic treatment (19–21), and a decrease in the level of persisters in a strain with deletions of 10 TA systems (23). Additional experiments led to a plausible model of persister formation in *E. coli*, as follows: starvation → RelA/SpoT → ppGpp → PPX (inhibition) → polyphosphate → Lon activation → antitoxin degradation → toxin release → inhibition of translation → drug-tolerant persister (21). This model has been widely accepted (27–31).

We sought to determine whether various stresses can induce the expression of mRNA interferases and persister formation. Our results were unexpected: at least some of the TAs were expressed under each stress tested (stringent response, osmotic stress, pH stress, and NaCl stress), but only the stringent response led to TA-dependent persister formation, and the phenotype is specific to fluoroquinolones—there was no effect on tolerance to ampicillin. We then decided to reexamine the role of these mRNA interferases in the antibiotic tolerance of persisters formed stochastically in unstressed culture and determine whether bacteria harbor an additional, overlooked mechanism of persister formation.

The *E. coli* Δ10TA mutant was reported to have a diminished level of persisters tolerant to unrelated antibiotics under common growth conditions in rich medium (23), and we confirmed this finding. However, in minimal medium, the Δ10TA mutation affected tolerance of ciprofloxacin but not of ampicillin. Another expectation of the mRNA interferase model of persister formation is the execution mechanism: the toxins degrade mRNA, which should inhibit translation, leading to dormant persisters. Surprisingly, we find that the Δ10TA mutation has the same effect on decreasing persister formation in the presence of chloramphenicol or tetracycline, which completely inhibit protein synthesis. How mRNA interferases are linked to persister formation remains unclear.

It is important to point out that a recent study reported that Δ10TA exhibits decreased resistance to ciprofloxacin (64). The ciprofloxacin MIC is the same in the wild type and Δ10TA, as determined by the standard broth microdilution method, which registers twofold changes. Using a more detailed range of concentrations, the authors report a 1.5-fold-higher ciprofloxacin MIC for the wild type than for Δ10TA (64). It is possible that this increased susceptibility accounts for the apparent decrease in tolerance of Δ10TA. The authors also report no difference in the levels of persisters of the wild type and Δ10TA surviving treatment with kanamycin, suggesting that interferase-type toxins may contribute to tolerance to some but not all antibiotics.

We next considered whether elements of a proposed “toxin activation pathway” could affect persister formation irrespective of TAs. It has been reported that ppGpp inhibits the PPX phosphatase and increased levels of polyphosphate activate the Lon protease that degrades the antitoxins. However, we find no phenotype in Δ*lon* Δ*sulA*, Δ*ppx*, Δ*ppk*, or Δ*ppx* Δ*ppk* mutants. This is consistent with similar negative findings from other recent publications (43–45).

Our study has also provided a serendipitous clue to a missing mechanism of persister formation in *E. coli*. One of the experiments that linked ppGpp to persisters was based on observing the antibiotic tolerance of individual cells stochastically expressing RpoS, which reports the levels of ppGpp (21). The authors argued that an increase in ppGpp will lead to degradation of antitoxin and release of active toxin. The cells expressing RpoS also had low levels of transcription of the ribosomal promoter *rrnB* P1, which is repressed by ppGpp. We reexamined this by sorting cells carrying *rrnB* P1-*gfp* in the Δ10TA and Δ*relA* Δ*spoT* backgrounds. Dim cells were enriched in persisters in all strain backgrounds, showing that *rrnB* P1 can report persisters through an additional mechanism.

Having established that the *rrnB* P1 promoter can report persister status independently of ppGpp, we considered its other known effector, ATP. The activity of *rrnB* P1 is positively controlled by ATP (47, 48), and that is apparently why sorting dim cells in an *rrnB* P1-*gfp* strain enables the isolation of persisters. Interestingly, RpoS is also an ATP reporter (65). Proteolysis of RpoS by ClpXP is inhibited at lower ATP levels. It appears that both persister reporters are coregulated by ATP and ppGpp. We also find that the fraction of such dim cells increases as the culture progresses from early exponential to stationary state, matching the known phenomenon of persister increase with cell density (53). It appears that persisters in a growing culture are cells that entered into a stationary-like state.

**FIG 5:**
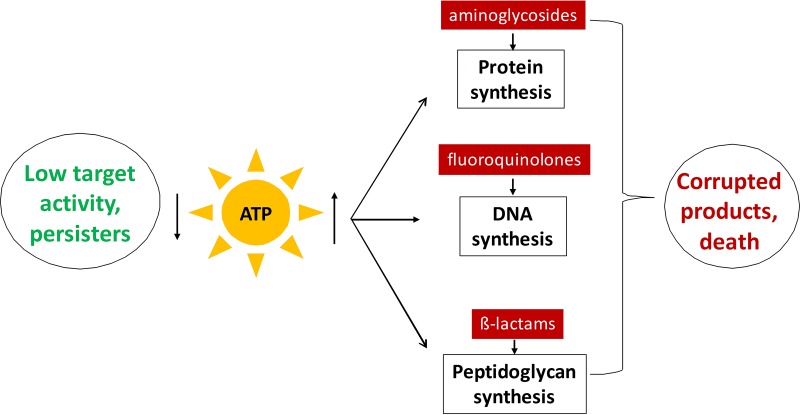
Proposed model for persister formation. Antibiotics kill by corrupting active cellular processes that require ATP. A decrease in ATP diminishes the target activity and leads to antibiotic tolerance.

ATP indeed seems like a good candidate for a general cause of tolerance, since most bactericidal antibiotics kill by corrupting active, energy-dependent targets. Fluoroquinolones act by irreversibly stabilizing gyrase-DNA intermediates that collide with replication forks, releasing lethal double-strand DNA breaks (52); aminoglycosides cause mistranslation, which produces toxic misfolded peptides damaging the membrane (51); and β-lactams kill cells by forcing a futile cycle of peptidoglycan synthesis (66). All of the antibiotic targets require ATP to function, and a drop in ATP will lead to decreased activity, resulting in drug tolerance. One well-known example is tolerance of β-lactams. Once a culture stops growing, peptidoglycan synthesis ceases, and cells become completely tolerant to cell wall-acting antibiotics (50). Apart from this well-understood mechanism of tolerance, we found that lowering intracellular ATP in a growing population to stationary levels with arsenate treatment strongly increases the level of persisters tolerant to fluoroquinolones. The same treatment dramatically decreases the rate of translation and diminishes DNA fragmentation caused by fluoroquinolones, explaining the mechanism of tolerance (Fig. 5). Taken together, our results suggest that the variation in the level of ATP serves as a mechanism of persister formation in *E. coli*.

It is notable that we recently found that ATP depletion appears to be the main mechanism of persister formation in *S. aureus* (32). Neither the stringent response nor toxin-antitoxin modules play a role in *S. aureus* persisters (32). ATP depletion may be a general mechanism of persister formation in bacteria.

While tolerance by a drop in the energy level explains persister formation, why some cells in a growing culture will have less ATP remains to be established. One possibility is that unavoidable stochastic variations in gene expression prevent rare cells from readjusting their metabolism to deteriorating conditions as the density of the culture rises, leading to a drop in ATP and drug tolerance. From this perspective, there is no specialized mechanism of stochastic persister formation. This random error hypothesis (although without the ATP component) was proposed by Vázquez-Laslop and coauthors (67). The findings of this study suggest an answer to the long-existing puzzle of stationary-phase populations forming very high levels of persisters. A drop in ATP in stationary-phase explains increased tolerance.

It appears that there are at least two different types of mechanisms that lead to persister formation in *E. coli* and probably in other bacteria as well: dedicated persister components, such as mRNA interferases, TisB, or the gain-of-function mutations of HipA, and a decrease in ATP, possibly caused by random errors or a low energy state of the population. It is important to note that one specialized mechanism of persister formation, the induction of the TisB toxin by the SOS response, leads to a drop in proton motive force, ATP, and drug tolerance (16, 17). The mechanism by which mRNA interferases cause tolerance seemed obvious, since overexpression of this type of toxins inhibits translation (40). However, we find that a drop in the level of persisters is the same in the presence or absence of protein synthesis inhibitors in a strain with deletions of 10 TAs. Notably, ectopic overexpression of *mazF* causes a futile cycle of RNA degradation/synthesis, which leads to a decrease in the energy level and drug tolerance (68). If stochastic activation of a given mRNA interferase toxin is enough to similarly produce a futile cycle, this would contribute to the general mechanism of persister formation by ATP depletion.

## MATERIALS AND METHODS

### Bacterial strains and growth conditions.

The strains used in this study are listed in Table S1 in the supplemental material. The methods used to generate mutants are described in Text S1. *E. coli* MG1655 and its derivatives were cultured in Luria-Bertani broth or morpholinepropanesulfonic acid (MOPS) minimal medium, supplemented with 0.2% glucose as specified in each figure. Bacteria were routinely grown at 37°C at 220 rpm. The medium was supplemented with kanamycin at 25 µg/ml to maintain plasmids where necessary.

10.1128/mBio.02267-16.8 TABLE S1  Strains used in this study. Download TABLE S1, DOCX file, 0.01 MB. Copyright © 2017 Shan et al. 2017 Shan et al. This content is distributed under the terms of the Creative Commons Attribution 4.0 International license.

10.1128/mBio.02267-16.9 TABLE S2  Primers used in this study. Download TABLE S2, DOCX file, 0.01 MB. Copyright © 2017 Shan et al. 2017 Shan et al. This content is distributed under the terms of the Creative Commons Attribution 4.0 International license.

10.1128/mBio.02267-16.10 TEXT S1  Supplemental materials and methods. Download TEXT S1, DOCX file, 0.02 MB. Copyright © 2017 Shan et al. 2017 Shan et al. This content is distributed under the terms of the Creative Commons Attribution 4.0 International license.

### Stress induction, TA expression, and survival measurement.

Bacterial strains were grown under stress conditions and compared to the same strains grown under nonstress conditions; promoter activity and survival under exposure to antibiotics were measured. Stress was induced as described in the following sections.

### (i) Osmotic stress.

Cultures were grown to early exponential phase in MOPS minimal medium. Water (control) or sucrose to 330 mM (stress) was added (69).

### (ii) Isoleucine starvation.

Cultures were grown in MOPS medium supplemented with all 20 amino acids with a final concentration of 400 μM isoleucine (control) (70), or the isoleucine concentration was reduced to 60 μM (stress) (39). An additional 100 mM MOPS adjusted to pH 7 was added to the medium to prevent pH change.

### (iii) Acid stress.

Cultures were grown in either unbuffered LB medium (control) or with the addition of 100 mM morpholineethanesulfonic acid (MES) adjusted to pH 5 (stress). Ciprofloxacin is sensitive to low pH, so ciprofloxacin tolerance was not assayed under acid stress.

### (iv) Phosphate starvation.

Cultures were grown in MOPS minimal medium with KH_2_PO_4_ either at 1.32 mM (control) or at a 10-fold-lower concentration for the phosphate-limiting medium (stress) (71).

### (v) Sodium stress.

Cultures were grown in MOPS minimal medium to early exponential phase, and then an additional 300 mM NaCl was added (stress). The control cultures had no additional NaCl (69).

### Promoter induction measurement.

MG1655 strains harboring *gfp* promoter plasmids from the *E. coli* promoter library were used for TA stress induction measurements (38). When an interferase promoter was not present in the library, a plasmid was constructed using the same method as described previously (38). All plasmids were confirmed by sequencing. The *gfp*-promoter strains were grown in 96-well plates in a fluorimeter at 37°C under the conditions described above. The medium was supplemented with kanamycin (25 μg/ml) to maintain plasmids. Absorbance at an optical density of 600 nm (OD_600_) and GFP fluorescence (emission 528 nm and excitation 485 nm) were measured every 30 min. The GFP value was background subtracted using a strain carrying a plasmid expressing promoterless *gfp*. To correct for the effect of slowed growth due to stresses, OD versus GFP values were plotted. Each GFP fluorescence value that fell within a 0.1-OD-unit bin for stress and nonstress conditions was compared. The average of all GFP values obtained under stress was compared with the average of all GFP values obtained under nonstress conditions. It was determined that a toxin had increased expression under a stress if there was a statistically significant (*P* < 0.05 by Student’s *t* test) increase of GFP fluorescence of at least 50% during three independent experiments. The analysis was limited to early exponential phase (OD of <0.5), to correspond to the time when antibiotics were added in the antibiotic survival assays.

### Antibiotic survival assay.

Bacteria were inoculated at 1:100 into LB medium or 1:50 into MOPS-based medium from an overnight culture. Cell cultures were grown for 2 h to 3.5 h to reach approximately the same CFU count (1 × 10^8^ to 4 × 10^8^ CFU/ml). Ampicillin (100 µg/ml) or ciprofloxacin (concentration as indicated) was added. At each time point after the addition of antibiotic, cultures were washed with 1% NaCl and plated on LB agar for CFU counts. The percent survival was calculated as follows: (final CFU/CFU at 0 h) × 100. The results are presented as the average results from at least 6 biological replicates, and error bars in the figures represent standard deviations. *P* values were calculated by two-tailed Student’s *t* test, and *P* values of less than 0.05 are considered significant. In the arsenate or chloramphenicol pretreatment antibiotic survival assays, the methods followed were as described above except that cultures were pretreated with arsenate (30 min) or chloramphenicol (45 min) before the addition of antibiotics.

### Flow cytometry analysis and cell sorting.

The fluorescent protein levels were analyzed with a BD Aria II flow cytometer (BD Biosciences) with a 70-μm nozzle. The cell populations were detected using forward and side scatter (FSC and SSC) parameters, and fluorescence was analyzed with an emitting laser of 488 nm and bandpass filter of 525/15 nm. For each sorting experiment, cells were treated with antibiotic as indicated in Fig. 2. For each culture, 32,000 events were collected from each fraction and dispensed directly to agar plates. The survival rate was calculated as CFU divided by total cells sorted to the plate. The results for survival are presented as the average results from at least 6 biological repeats, and error bars in the figures represent standard deviations. *P* values were calculated by the Student *t* test, and *P* values smaller than 0.05 are considered significant. Data were acquired using FACSDiva software, and graphs were generated by FlowJo (Tree Star software).

### ATP measurement.

The ATP levels of stationary and exponential cultures with the addition of various concentrations of arsenate were measured using a BacTiter Glo kit (Promega) according to the manufacturer’s instructions. Background ATP was subtracted using spent medium from each condition.

### TUNEL assay.

The TUNEL assay was performed as previously described, following the instructions in the *in situ* cell death detection kit (Roche) (57). Briefly, after antibiotic treatment, ~1 × 10^8^ cells were pelleted and washed with phosphate-buffered saline (PBS). The pellets were then resuspended into 500 µl of ice-cold fixing solution (4% paraformaldehyde in PBS). After 30 min of incubation on ice, the cells were pelleted, washed, and incubated in 500 μl of ice-cold permeabilization solution (0.1% Triton X-100 and 0.1% sodium citrate) for 2 min. The cells were pelleted, washed, and incubated in 50 μl of TUNEL reaction mixture, including fluorescein isothiocyanate (FITC)-dUTP and deoxynucleotidyl transferase enzyme, at 37°C in the dark for 1 h. Samples were washed and then resuspended in PBS for FACS analysis. The FITC signal was analyzed with an emitting laser at 488 nm and bandpass filter of 525/15 nm using a BD Aria II flow cytometer (BD Biosciences) with a 70-μm nozzle.
