# Supplementary figures and images for: ATP-Dependent Persister Formation in Escherichia coli
Source: mBio. 2017 Feb 7;8(1):e02267-16. doi: 10.1128/mBio.02267-16 (PMC5296605; doi:10.1128/mBio.02267-16)

## Slide 1
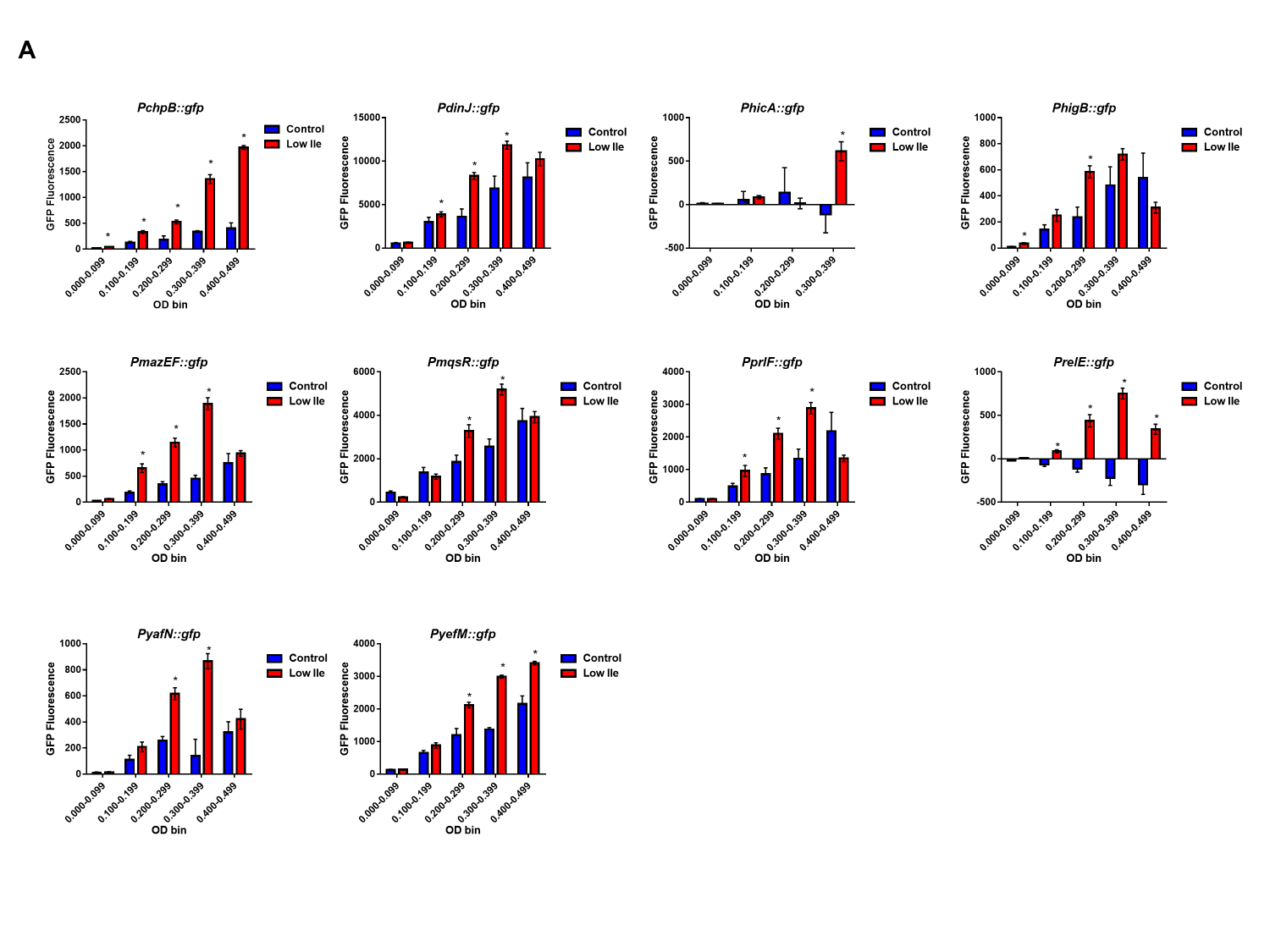

## Slide 2
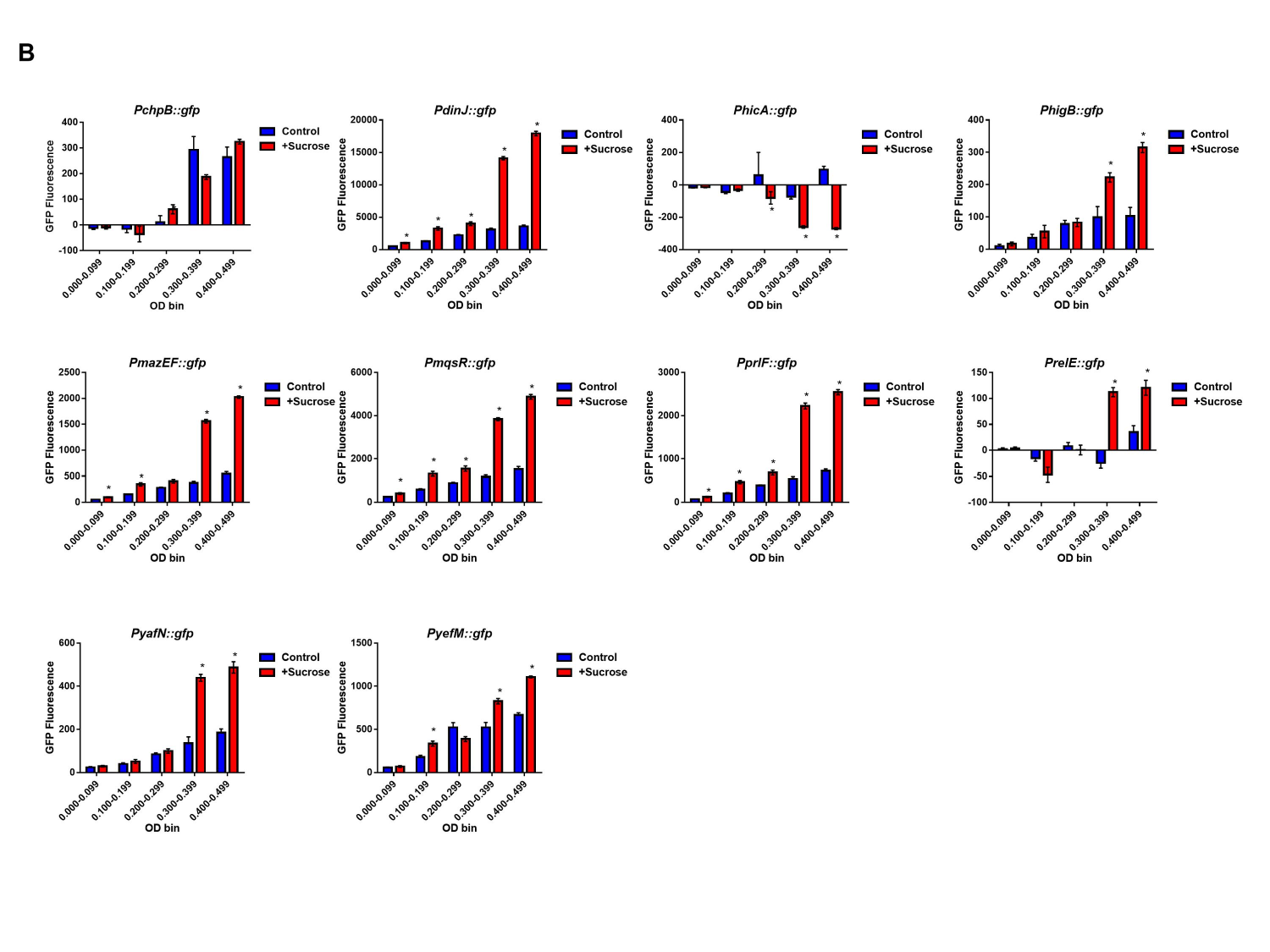

## Slide 3
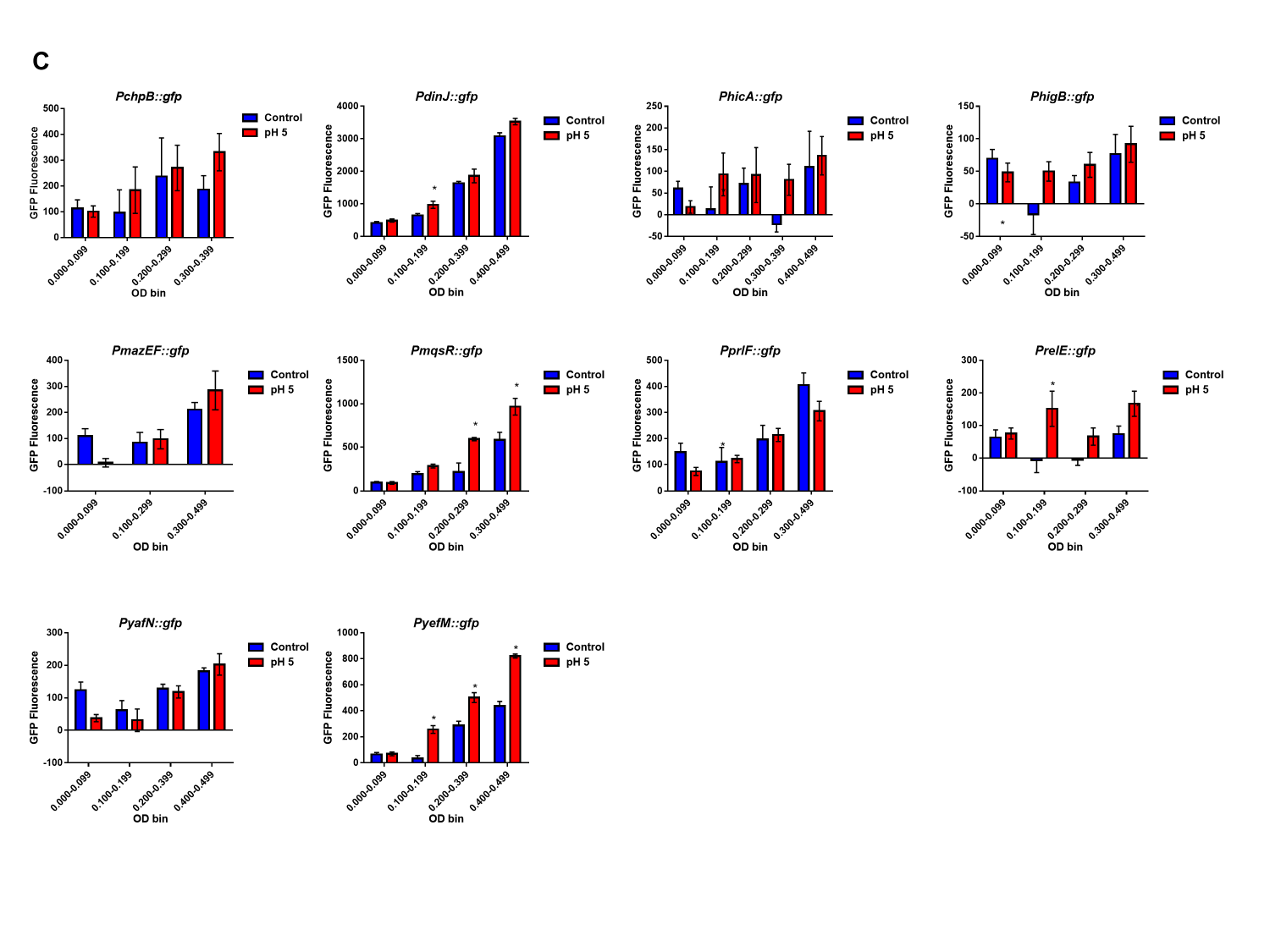

## Slide 4
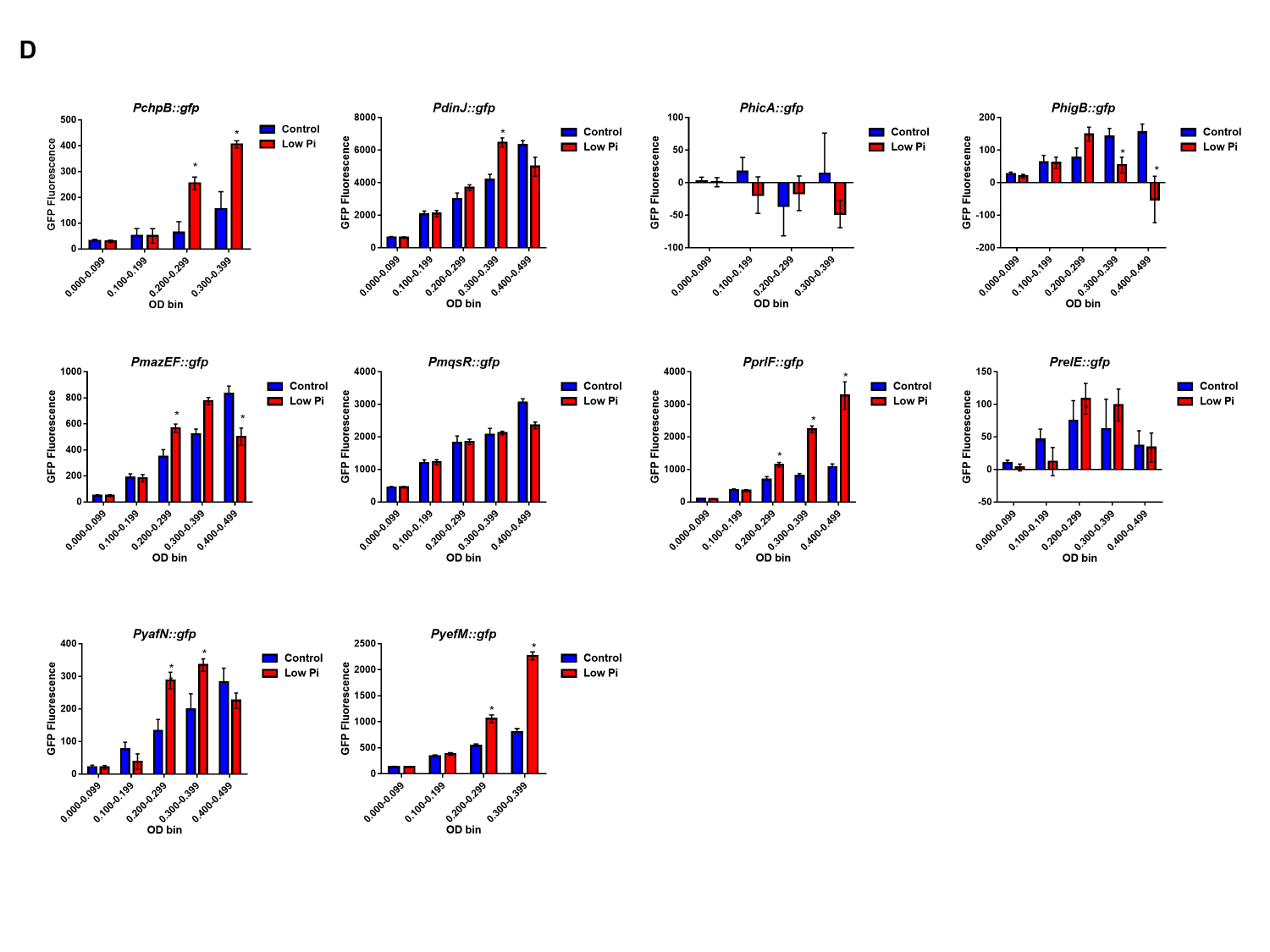

## Slide 5
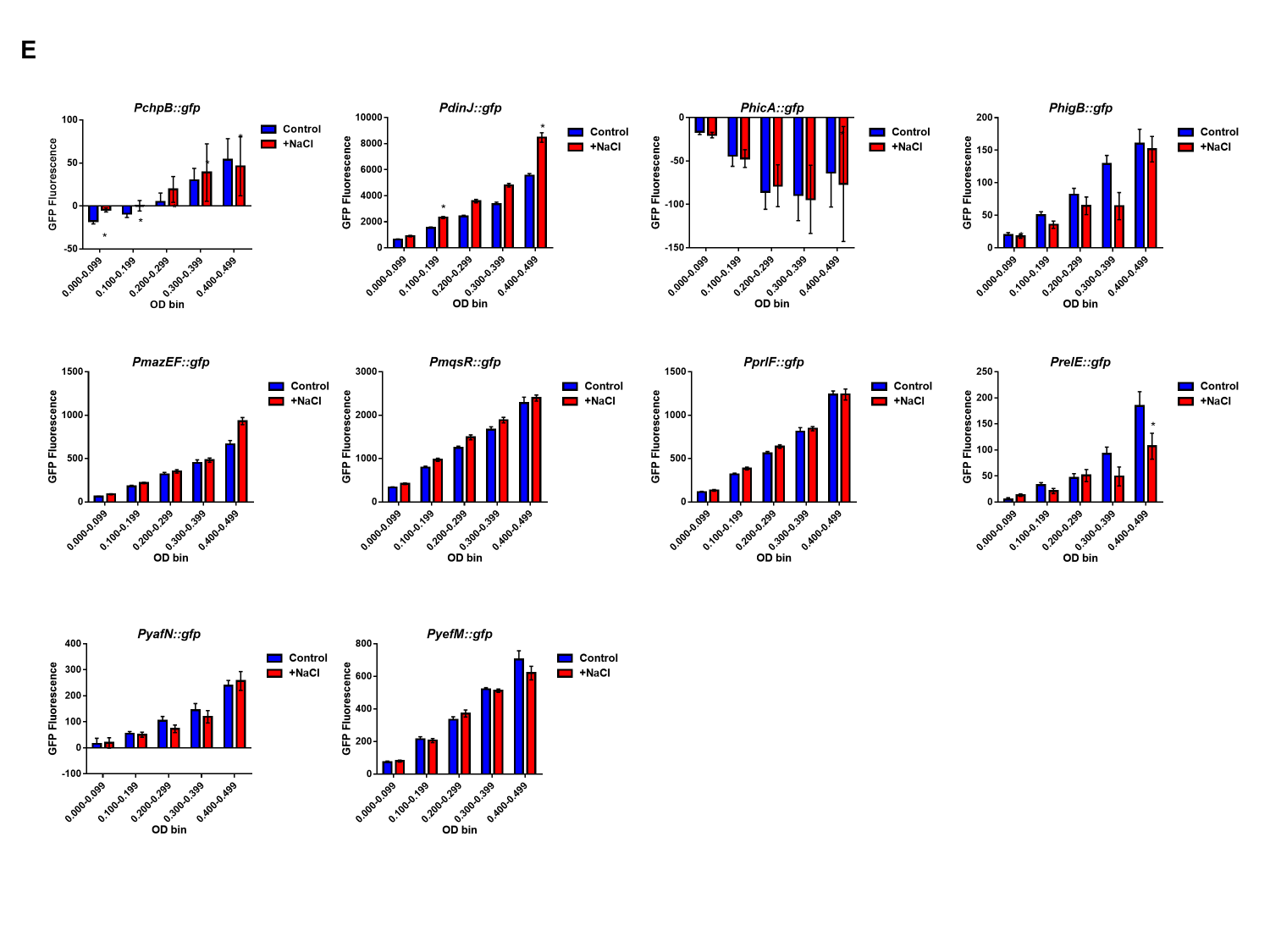

Supplement: FIG S1 [file mbo001173179sf1.ppt]

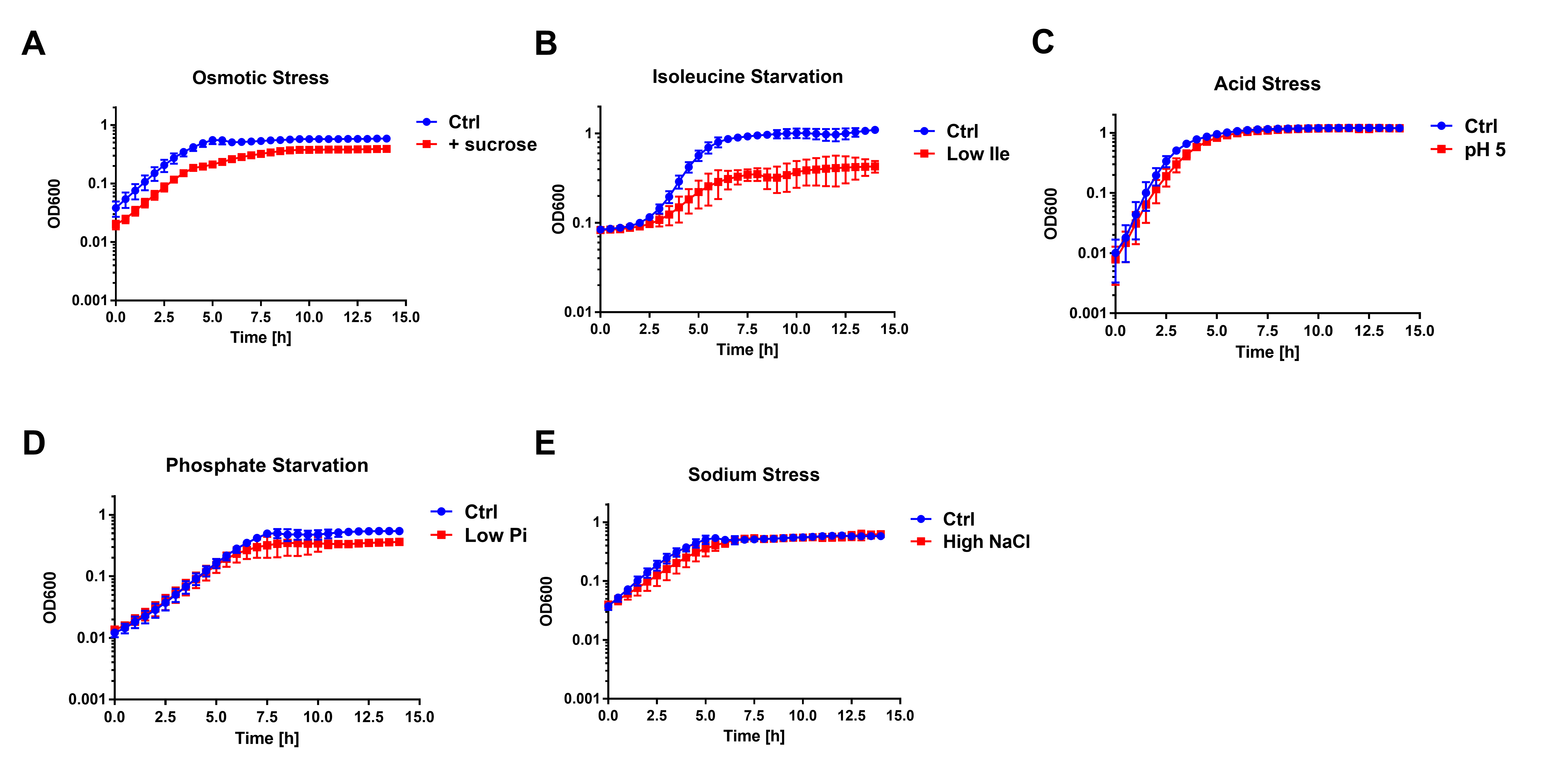

Supplement: FIG S2 [file mbo001173179sf2.tif]

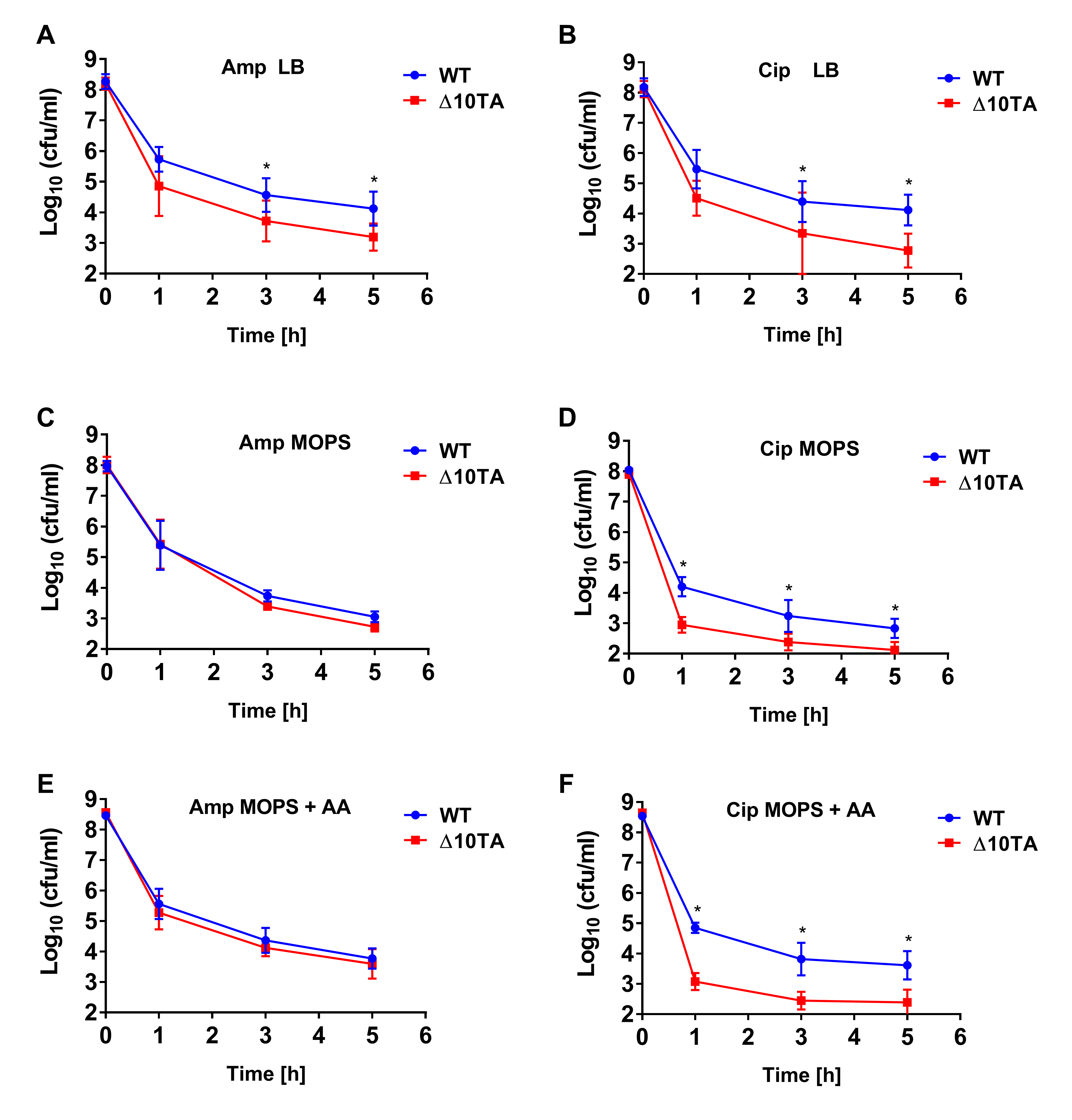

Supplement: FIG S3 [file mbo001173179sf3.tif]

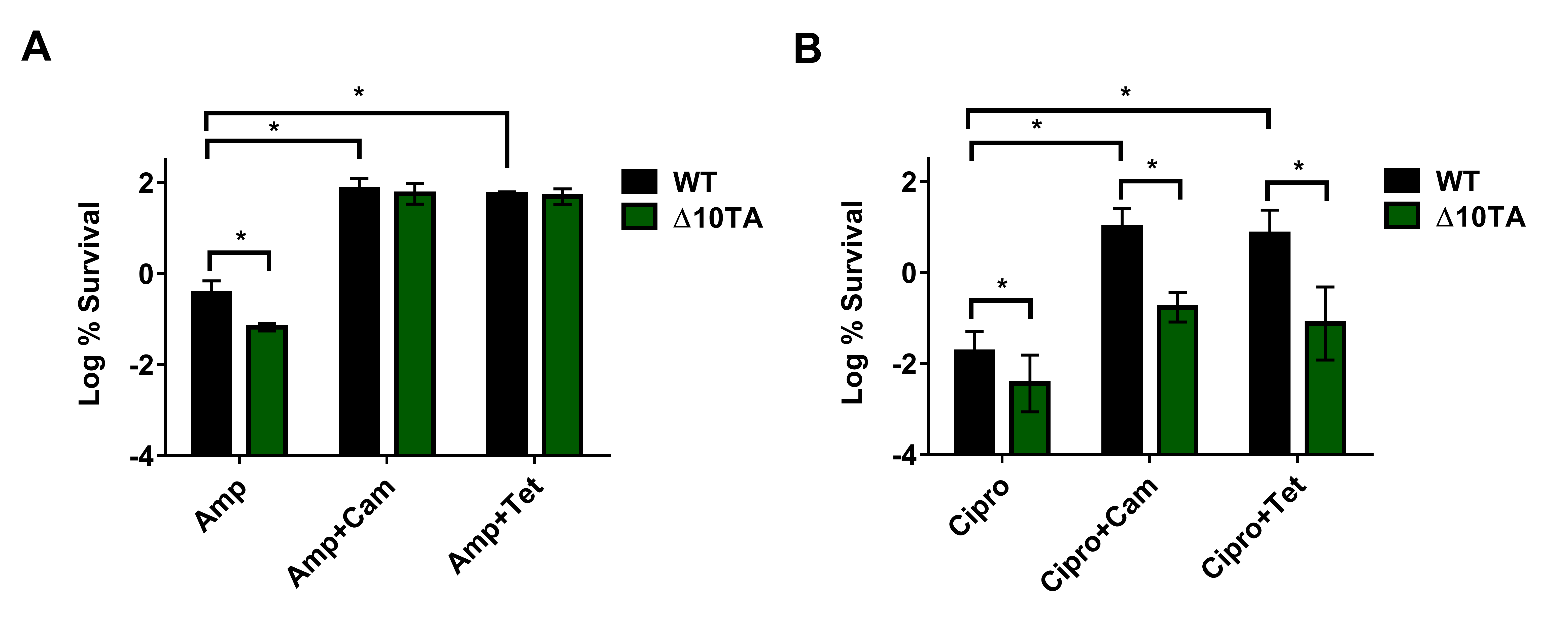

Supplement: FIG S4 [file mbo001173179sf4.tif]

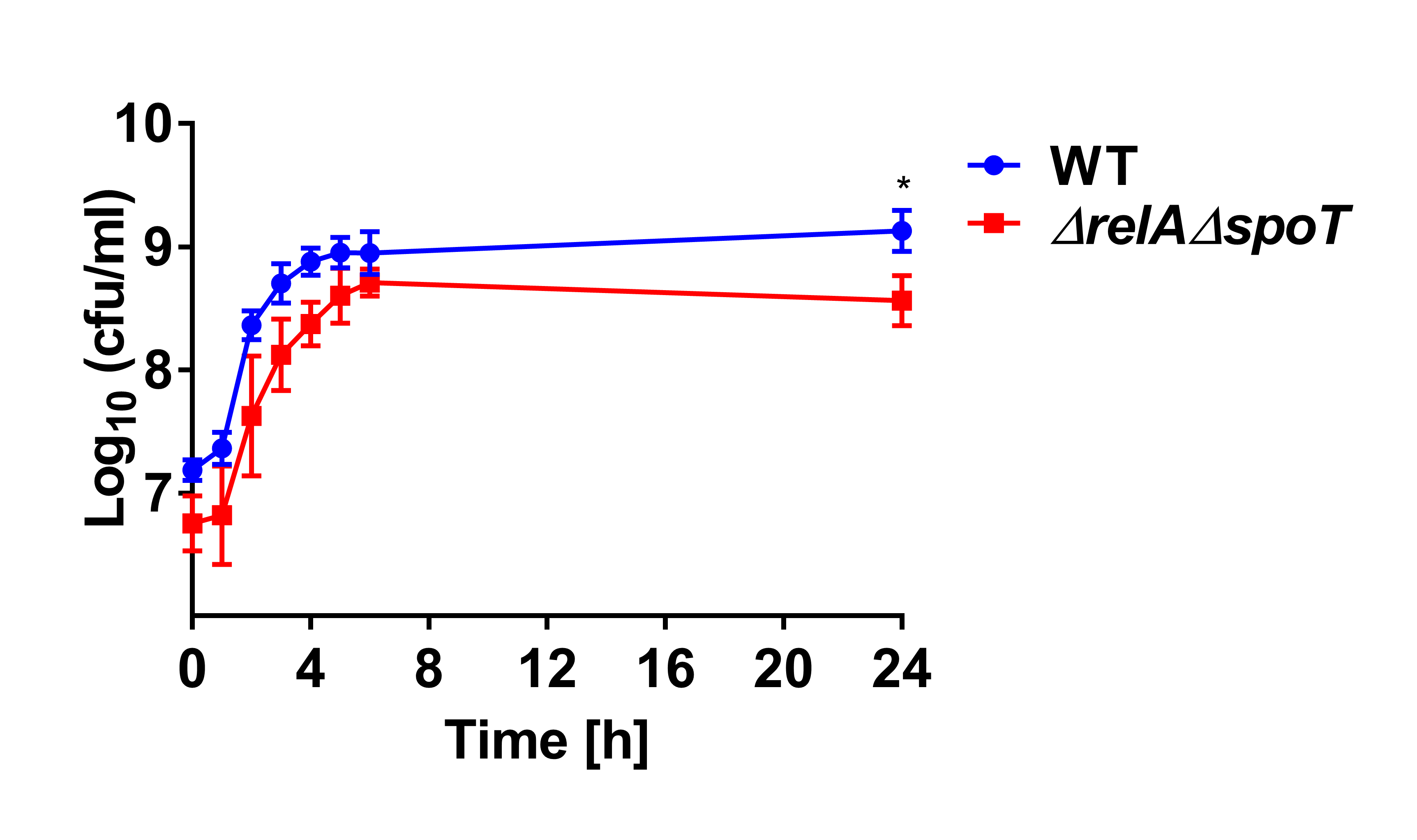

Supplement: FIG S6 [file mbo001173179sf6.tif]
