# Supplementary material for: ATP-Dependent Persister Formation in Escherichia coli
Source: mBio. 2017 Feb 7;8(1):e02267-16. doi: 10.1128/mBio.02267-16 (PMC5296605; doi:10.1128/mBio.02267-16)
Supplement: TABLE S1 [file mbo001173179st1.docx]

**Table S1 Strains used in this study**

| Strain | Reference |
| --- | --- |
| MG1655 (WT) |  |
| MG1655 pUA66 (promoterless *gfp*) | (38) |
| MG1655 pUA66 P*_chpSB_*::*gfp* | This work |
| MG1655 pUA139 P*_dinJyafQ_*::*gfp* | (38) |
| MG1655 pUA66 P*_hicAB_*::*gfp* | This work |
| MG1655 pUA139 P*_higAB_*::*gfp* | (38) |
| MG1655 pUA139 P*_mazEF_*::*gfp* | (38) |
| MG1655 pUA139 P*_mqsRA_*::*gfp* | (38) |
| MG1655 pUA66 P*_prlFyhaV_*::*gfp* | (38) |
| MG1655 pUA66 P*_relBE_*::*gfp* | (38) |
| MG1655 pUA66 P*_yafNO_*::*gfp* | (38) |
| MG1655 pUA139 P*_yefMyoeB_*::*gfp* | This work |
| Δ*yafQ::frt* | This work |
| Δ10TA | (23) |
| Δ*lon* | (21) |
| Δ*lon*Δ*sulA* | (21) |
| Δ*ppx::frt* | This work |
| Δ*ppk::frt* | This work |
| Δ*ppk ppx::cam* | This work |
| Δ*relA::frt* Δ*spoT::frt* | This work |
| MG1655 *rrnB* P1::*gfp*GFP^unstable^(ASV) | (21) |
| ASV Δ10TA | This work |
| ASV Δ*relA::frt* Δ*spoT::frt* | This work |
| ASV *ΔpurE::km* | This work |
| ASV Δ*relA::frt* Δ*spoT::frt ΔpurE::km* | This work |
