# Supplementary material for: ATP-Dependent Persister Formation in Escherichia coli
Source: mBio. 2017 Feb 7;8(1):e02267-16. doi: 10.1128/mBio.02267-16 (PMC5296605; doi:10.1128/mBio.02267-16)
Supplement: TABLE S2 [file mbo001173179st2.docx]

**Table S2. Primers used in the study.**

| Primer name | Sequence |
| --- | --- |
| spoT del 1 | TTGTATCTGTTTGAAAGCCTGAATCAACTGATTCAAACCTACCTGCCGGATTGTGTAGGCTGGAGCTGCTTCGA |
| spoT del 2 | TTAATTTCGGTTTCGGGTGACTTTAATCACGTCTGGCATCACGCGGATTCATATGAATATCCTCCTTAGTTC |
| ppkx_del1 | GCCATAATATCCAGGCAGTGTCCCGTGAATAAAACGGAGTAAAAGTGGTATTGTGTAGGCTGGAGCTGCTTCGA |
| ppkx_del2 | GAAAGTGCCTGAATAATGCGGGCCGACATTTCTCGTCGGCCCGCAAAGTACATATGAATATCCTCCTTAGTTC |
| pUA139 P_yefM_::GFP 1 | GTAGTAGGATCCCAGGATGATGGTGATGATGG |
| pUA139 P_yefM_::GFP 2 | GTAGTACTCGAGTTGTTGCCGACAAATTCTGA |
| pUA66  P_chpS_::GFP 1 | GTAGTACTCGAGTAATTGAAAGCGGCGACTG |
| pUA66  P_chpS_::GFP 2 | GTAGTAGGATCCGGGAATGACCATACCTGCAC |
| pUA66  P_hicA_::GFP 1 | GTAGTACTCGAGACCGATAACGCCTTCGTAAA |
| pUA66  P_hicA_::GFP 2 | GTAGTAGGATCCCTACATCGACGCCCTGAGAT |
