# Supplementary material for: ATP-Dependent Persister Formation in Escherichia coli
Source: mBio. 2017 Feb 7;8(1):e02267-16. doi: 10.1128/mBio.02267-16 (PMC5296605; doi:10.1128/mBio.02267-16)
Supplement: TEXT S1 [file mbo001173179s1.docx]

**Supplemental Materials and Methods**

Single deletion mutants were constructed using P1 transduction from the KEIO collection of *E. coli* deletion strains into MG1655 ([1](#_ENREF_1)). The kanamycin cassette was cured using the Flp recombinase system on pCP20 ([2](#_ENREF_2)).

The *relAspoT* deletion mutant was constructed by first transducing the *relA* deletion allele from the KEIO collection as described above into MG1655. A clean deletion of *spoT* was made using the red recombinase methods described by Datsenko and Wanner and removing the antibiotic cassette with pCP20 ([3](#_ENREF_3)).

The *ppx/ppk* deletion mutant was constructed using the lambda red recombinase method described by Datsenko and Wanner ([3](#_ENREF_3)).

The *rrnB* P1::*gfp*^unstable^ strains were constructed by P1 transduction from the donor strain ASV.

Strains used in this study are listed in Table S1. Oligonucleotide primers used in this study are listed in Table S2.

Whole genome sequencing is performed on Illumina Hi-Seq 2500 platform using single-end 50 bp sequencing. Both ∆10TA and the parental MG1655 sequencing data was mapped to E. coli MG1655 reference genome by CLC genomic bench 9 (Qiagen). SNP was detected with minimum coverage of 150 reads and minimum frequency 50% for both strains and was compared. SNPs in ∆10TA comparing to the parental strain was listed in Supplemental Dataset 1.

Supplemental References

1. Baba T, Ara T, Hasegawa M, Takai Y, Okumura Y, Baba M, Datsenko KA, Tomita M, Wanner BL, Mori H. 2006. Construction of *Escherichia coli* K-12 in-frame, single-gene knockout mutants: the Keio collection. Mol Syst Biol 2:2006.0008.

2. Cherepanov PP, Wackernagel W. 1995. Gene disruption in *Escherichia coli*: TcR and KmR cassettes with the option of Flp-catalyzed excision of the antibiotic-resistance determinant. Gene 158:9-14.

3. Datsenko KA, Wanner BL. 2000. One-step inactivation of chromosomal genes in *Escherichia coli* K-12 using PCR products. Proc Natl Acad Sci U S A 97:6640-6645.
